# Cases of BA.2.75 and recent BA.2.75.2 subvariant of Omicron are increasing in India: Is it alarming at the global level?

**DOI:** 10.1016/j.amsu.2022.104963

**Published:** 2022-11-18

**Authors:** Chiranjib Chakraborty, Manojit Bhattacharya, Kuldeep Dhama

**Affiliations:** Department of Biotechnology, School of Life Science and Biotechnology, Adamas University, Kolkata, West Bengal, 700126, India; Department of Zoology, Fakir Mohan University, Vyasa Vihar, Balasore, 756020, Odisha, India; Division of Pathology, ICAR-Indian Veterinary Research Institute, Izatnagar, Bareilly, 243122, Uttar Pradesh, India

Dear Editor

Scientists have noted several variants of SARS-CoV-2 during the pandemic's last two and half years. Ongoing random mutations in SARS-CoV-2 have generated these variants. The World Health Organization (WHO) provided naming of the variants using Greek alphabets along with the existing Pango nomenclature for SARS-CoV-2 (a dynamic nomenclature of the virus lineage). Some examples are B.1.1.7 variant as Alpha (α), B.1.351 variant as Beta (β), B.1.617.2 variant as Delta (δ), and P.1 as Gamma (γ), etc. It has been noticed that these variants had created a surge in COVID-19 cases in the country of origin and then rapidly transmitted to different countries [1,2]. However, among these variants, some variants are noted as extensively transmitted variants. The last SARS-CoV-2 variant, Omicron, is observed as a highly transmitted variant.

The Omicron variant first emerged in South Africa. The variant was identified in November 2021. WHO intimated about this new variant on November 24, 2021 [3]. After identifying the variant, it has been noted that it has a high transmission capacity as well as ability to overpower vaccine induced protective immunity and antibodies-based therapies via immune escape mechanisms. Due to the high transmission rate, the WHO labeled the new variant as variant of concern (VOC) within two days [4]. Subsequently, the Omicron variant has now spread to about 150 countries. Along with the high transmission rate, numerous mutations are noted in the genome of the variant [5,6]. Approximately 50 mutations have been revealed throughout the genome of Omicron. At the same time, it was recorded that the hotspot of SARS-CoV-2 mutations is the ‘S’ protein. Scientists noticed that the Omicron spike protein harbors approx. 32 mutations in the sequence [6]. About 26 mutations are noted as RBD (receptor binding domain) part of the ‘S’ protein sequence [7]. Analysis of the genome sequences from the different countries revealed that the ongoing random mutating process in the Omicron genome has created several sublineages or subvariants of this variant from time to time.

From the surveillance studies of the Omicron variant, more than one hundred subvariant or sublineages have been reported to be generated from Omicron. Among them, the emerging sublineages are BA.1, BA.2, BA.4 and BA.5 [8,9]. The BA.2 subvariant was noted during June 2022 in European countries. After that, several subvariants were created from BA.2. Some subvariants are BA.2.74, BA.2.75, BA.2.76, etc. [10]. However, BA.2.75 and BA.2.12.1 are significant subvariants of BA.2 (Fig. 1a).

**Fig. 1:**
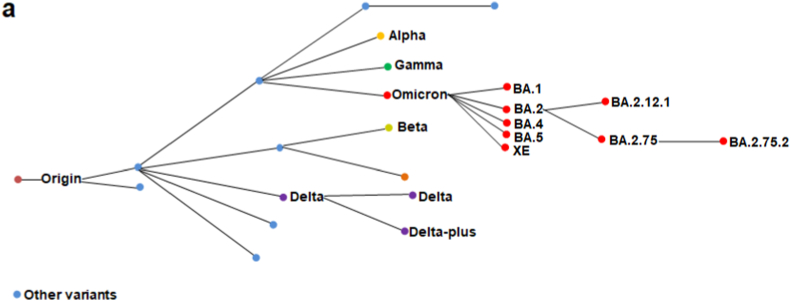

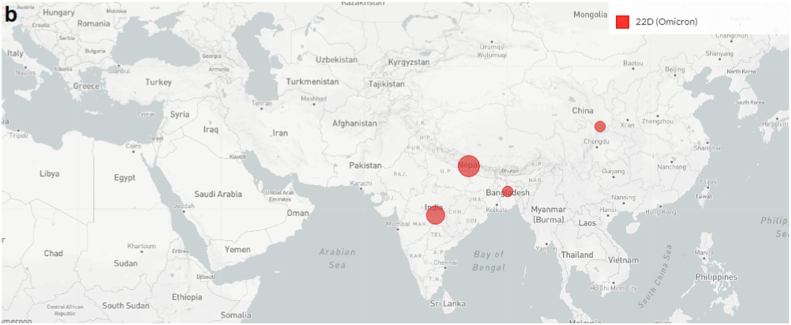

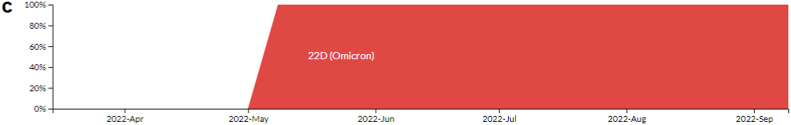

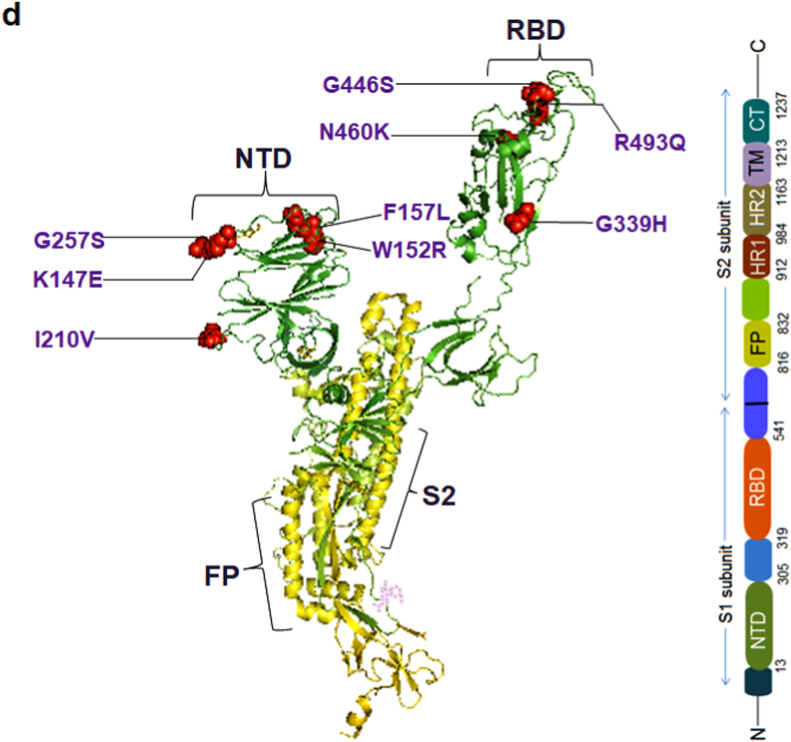
The origin, distribution, frequencies, and significant mutations of BA.2.75 (a) A schematic diagram shows the origin and evolution of BA.2.75 or BA.2.75.1 (b) The map shows the distribution of BA.2.75 (c) The diagram shows the frequencies of BA.2.75. The diagram indicated that the subvariant started to increase from May 2020 (d) A schematic diagram shows the significant S-protein mutations of BA.2.75. Fig.1b and 1c were developed using the Nextstrain server.

Presently, BA.2.75 was identified in about 20 countries worldwide. Suddenly, from the surveillance of the genome sequences of SARS-CoV-2 variants, scientists noted that BA.2.75 is quickly rising in India. Although the BA.2.75 cases are presently low in other countries compared to India, however, scientists have alarmed other countries regarding the possible risk of rise of BA.2.75 instances. At the same time, scientists noted that the number of other subvariants of Omicron is falling remarkably in many countries. Nevertheless, hospitalization is low due to the infection of the BA.2.75. It has been found that the maximum number of samples from India contain the genome of BA.2.75 subvariant. Scientists are assuming that BA.2.75 will keep growing globally, mainly in Asia [11]. The BA.2.75 was first identified in India, a specimen collected from India on January 7, 2022 revealed the presence of BA.2.75 (Fig. 1a) [12]. Afterwards, this subvariant was identified in several other countries, and a gradual increase in the subvariant was noted in some countries from May–June 2022 (Fig. 1c). The subvariant was noted in countries like the USA, Canada, the UK, Singapore, and Japan [12]. A number of properties were characterized for the BA.2.75 subvariant by quite a few scientists. In a simulation study, Zappa et al. reported that, compared to the BA.5 variant, BA.2.75 showed about 57-fold increased receptor binding affinity (ACE2 receptor). The subvariant also showed markedly higher receptor binding affinity (more than 3000-fold) compared to the Alpha (B.1.1.7) variant [13]. Shaheen et al. defined the BA.2.75 subvariant with the spike protein mutations: the R493Q, G446S, W152R, and K147E. They also reported that R493Q and G446S are alarming mutations. The G446S mutation might have a role in immune resistance or ACE2 receptor binding [10]. Recently, Sheward et al. illustrated that nine additional mutations are found in the spike protein of BA.2.75 compared to BA.2, which are R493Q, N460K, G446S, G339H, G257S, I210V, F157L, W152R, and K147E (Fig. 1d). The subvariant has shown the immune escape of neutralizing antibodies (nAbs). Sheward et al. also stated that the G446S mutation of BA.2.75 has been responsible for nAbs escape phenomena which are elicited by current COVID-19 vaccines. Furthermore, the mutation site is responsible for the immune evasion of the LY-CoV140 antibody (bebtelovimab). At the same time, a decrease in antibody potentiality against BA.2.75 subvariant has been reported. Scientists have also observed the potency of cilgavimab antibody to be reduced 11-fold against BA.2.75 compared to B.1 variant (D614G). At the same time, It has also been reported that several antibodies were unable to neutralize BA.2.75. Such antibodies are etesevimab, bamlanivimab, imdevimab, and casivirimab. However, other than a minor reduction in potency of bebtelovimab, it retains sensitivity against BA.2.75. Some other antibodies show moderate susceptibility to BA.2.75 subvariant, which are cilgavimab and tixagevimab [14].

It has been recently noted that BA.2.75 subvariant is further mutating and forming a new subvariant called BA.2.75.2 (Fig. 1a). The new subvariant BA.2.75.2 is currently reported in India. The local news agencies of India said the newer subvariant BA.2.75.2 is growing very quickly [15]. They have reported that this subvariant is more transmissible than others and trying to establish it as a dominant Omicron subvariant. However, there is no scientific report published till date in this direction. The BA.2.75.2 subvariant has now been present in eight countries including India, Chile, England, Singapore, Spain and Germany [15].

Global tracking of BA.2.75.2 is immediately required as earlier BA.2.75 replaced BA.4 and BA.5 subvariants, and now BA.2.75.2 is rather comparatively more transmissible, therefore could pose high chances to spread worldwide rapidly. Of note, further research is needed to understand the different properties of this newly emerged subvariant. At the same time, scientists should unfold the different significant features like transmission, antibody susceptibility, mutation pattern, receptor binding affinity, and immune evasion of the BA.2.75.2. It is also necessary to develop proper diagnostics for confirmatory detection of the BA.2.75.2 subvariant. We urge scientists to study more in this direction to solve the unfold properties of the BA.2.75.2 and enhance its surveillance and monitoring activities. This will further help us to fight against this highly contagious and feasibly more immune evasive subvariant, formulating proactive control measures and preparedness plans for avoiding any possible new wave of Omicron infections, and save the humanity amid the ongoing COVID-19 pandemic. Researchers have anticipated that newer subvariants might keep emerging in coming time, and this would pave ways for the COVID-19 pandemic to linger far into the future. Under such scenario, the presently available COVID-19 vaccines will become less potent to render protective immunity and thus will need to be updated. At the same time, the next generation vaccines including mutation proof vaccines will required to counteract the newly evolving variants or subvariants.

## Ethical approval

No applicable.

## Sources of funding

No fund received.

## Author contribution

**Chiranjib Chakraborty:** Conceptualization, Data Curation, Investigation, Writing - Original Draft, Writing - review & editing.

**Manojit Bhattacharya:** Validation and figure development.

**Kuldeep Dhama:** Validation; editing-reviewing.

All authors critically reviewed and approved the final version of the manuscript.

## Trial register number

1. Name of the registry: Not applicable
2. Unique Identifying number or registration ID: Not applicable
3. Hyperlink to your specific registration (must be publicly accessible and will be checked): Not applicable

## Guarantor

Professor Chiranjib Chakraborty,

Department of Biotechnology, School of Life Science and Biotechnology,

Adamas University, Kolkata, West Bengal 700126, India.

Email: drchiranjib@yahoo.com Tel: +91-9871608125

## Consent

Not applicable.

## Provenance and peer review

Not commissioned, internally peer-reviewed.

## Data statement

The data in this correspondence article is not sensitive in nature and is accessible in the public domain. The data is therefore available and not of a confidential nature.

## Declaration of competing interest

All authors report no conflicts of interest relevant to this article.
